# Hepatitis B virus, alcohol, and liver cancer

**DOI:** 10.3389/fonc.2026.1751474

**Published:** 2026-02-18

**Authors:** Anup S. Pathania, Natalia A. Osna

**Affiliations:** Department of Pharmacology and Experimental Neuroscience, University of Nebraska Medical Center, Omaha, NE, United States

**Keywords:** alcohol, HBV, hepatocellular carcinoma, immune dysregulation, liver inflammation, risk factors, viral replication

## Abstract

Viral hepatitis is an infection caused by hepatotropic viruses that leads to liver inflammation and may progress from a prolonged asymptomatic phase to decompensated liver disease. Among the main types, hepatitis A (HAV), B (HBV), and C(HCV) are most common, with HBV being the most widespread worldwide. Both HBV and HCV contribute substantially to global morbidity and mortality. However, HBV accounts for the most significant burden of chronic infections and associated complications, including cirrhosis and hepatocellular carcinoma (HCC). HBV infection is one of the leading causes of HCC globally. The chronically infected individuals face a lifetime risk of up to 25% of developing liver cancer, which progresses from cirrhosis and is affected by many comorbidities. Clinical and epidemiological studies, including research from our group, have shown that alcohol consumption in HBV-infected people speeds up disease progression, increases viral replication, and worsens liver damage. Heavy alcohol use is a major cofactor that significantly raises the risk of HCC in patients with HBV-related cirrhosis. Despite this well-established link, the safe level of alcohol consumption for HBV-infected patients remains unclear. Additionally, other cofactors such as viral co-infections, metabolic disorders, genetic predisposition, demographic factors, and environmental exposures interact with alcohol to influence HBV-related disease outcomes. Understanding the systemic effects of alcohol and its underlying mechanisms in HBV development is essential to defining its role as a comorbid factor in liver disease progression. This brief review highlights current knowledge and mechanistic insights into how alcohol influences HBV infection, contributes to HCC development, and acts as a comorbid factor that worsens disease severity.

## 1 Introduction

Hepatotropic viruses are a group of viruses that possess the unique ability to target and replicate in liver hepatocytes, inducing liver-related diseases (1). These viruses belong to different viral families, yet all share a specific affinity for the liver and cause hepatitis. They replicate primarily in hepatocytes and are the leading cause of liver inflammation. Each virus is taxonomically distinct, with a unique genomic structure and replication mechanism, reflecting its evolutionary diversity. The clinical manifestations linked to liver-specific disease result from their shared tropism.

The most common hepatotropic viruses are HAV, HBV, HCV, Hepatitis D virus (HDV), and Hepatitis E virus (HEV). HAV and HEV are generally acute and self-limiting. In contrast, HBV, HCV, and HDV are major causes of chronic liver disease, cirrhosis, and HCC. Among these, HBV is considered the most important due to its significant global health impact, including widespread chronic infection, high infectivity, and its role as a leading cause of liver disease and liver cancer (2). HBV is a partially double-stranded DNA virus from the Hepadnaviridae family. It is transmitted through blood, sexual contact, and perinatally from mother to child. While most adults clear acute HBV infection, about 5-10% progress to chronic hepatitis B (CHB) (3). In infants and young children, the risk of chronicity is significantly higher (up to 90%) (4). CHB can progress to cirrhosis, liver failure, and HCC, accounting for around 1 million deaths annually and contributing significantly to liver-related morbidity and mortality.

Despite the availability of a safe and effective vaccine, HBV remains a leading cause of chronic liver disease worldwide. Over 2 billion people have been infected with HBV, with about 296–300 million chronic carriers currently living with the infection. Antiviral therapies, such as nucleos(t)ide analogs and interferons, can control viral replication but rarely cure the infection due to the persistence of HBV covalently closed circular DNA (cccDNA) (5, 6). HBV remains a global health burden despite effective vaccines and potent antivirals. Its unique replication strategy, persistence of cccDNA, and immune evasion mechanisms make eradication difficult. In addition, certain risk factors exacerbate the clinical outcomes of HBV infection, with alcohol (ethanol) use being one of the most significant.

While HBV alone can drive CHB, cirrhosis, and HCC, concurrent alcohol use accelerates liver damage, increases HBV persistence, and raises the risk of malignancy. Studies show that heavy alcohol intake combined with chronic HBV infection dramatically raises the risk of cirrhosis and liver cancer compared to either factor alone. Even moderate alcohol intake increases oxidative damage and liver injury in HBV-infected patients (7). The interaction between HBV and alcohol is multifactorial, involving direct hepatotoxicity, immune and metabolic dysregulation, oxidative stress, and genetic alterations in viral replication. These combined effects cause the disease to progress quickly, emphasizing the importance of avoiding alcohol and maintaining good metabolic health in people with HBV infection.

## 2 Clinical evidence linking alcohol consumption to HBV-associated end-stage liver disease and HCC

Clinical studies indicate a strong association between HBV, alcohol, and HCC. Consuming more than 3 units/day (12-13 g alcohol/unit) increases the likelihood of cirrhosis in both men (OR 4.3) and women (OR 5.7), with a multiplicative interaction between alcohol intake and HBsAg positivity further elevating the risk (8). In HBV-related cirrhosis, the 5-year cumulative risk of HCC is 15% in high-endemic regions and 10% in Western populations. By contrast, alcoholic cirrhotics without viral infection have a lower 5-year HCC risk (8%), and advanced biliary cirrhosis carries a 4% risk (9).

The American College of Gastroenterology (ACG) strongly recommends that patients with CHB avoid alcohol. This guidance is supported by cohort studies showing that adults with HBV and chronic liver disease have higher odds of cirrhosis and increased HCC risk (8). Among cirrhotic patients, those with both HBV and alcohol use disorder (AUD) had the highest 10-year cumulative incidence of HCC (52.8%), compared with 39.8% in HBV alone and 25.6% in AUD alone. In a cohort of 1,515 cirrhotic patients, HBV patients with heavy chronic alcohol use (>80 g/day for over 5 years) had a significantly higher 10-year incidence of HCC than HBV cirrhotics without heavy alcohol use (HR 2.13, 95% CI 1.62-2.81, P <0.001) (10).

Similar findings were reported in an Australian population study. HBV patients hospitalized with alcoholic liver disease (ALD) alone, without cirrhosis, had a significantly increased HCC risk (HR ≈ 6.4). Cirrhosis alone had an even greater effect (HR ≈ 24.1). Patients with both HBV-related cirrhosis and ALD had the highest risk (HR ≈ 28.2), although the interaction was slightly less than additive. Age, male sex, and remoteness also influenced HCC risk. More substantial effects were observed in older populations (11). A recent meta-analysis that included over 33,000 HBV patients from 45 studies, mainly in Asia, found that alcohol increases the risk of both cirrhosis and HCC in a dose-dependent way. For every 12 grams (approx. one standard drink) of alcohol consumed daily, the risk of cirrhosis rises by about 6% and the risk of HCC by about 11% (12). Another study further showed that alcohol acts as an important cofactor in HBV-infected patients, even without cirrhosis. It increases the risk of both liver cancer and death (13).

Overall, although existing reports are limited, substantial global evidence highlights alcohol’s critical role in HBV pathobiology and its contribution to HBV-associated HCC. Despite effective antiviral therapies, alcohol abuse remains a significant factor that increases HCC risk and diminishes treatment efficacy. These findings underscore the importance of assessing and addressing alcohol use in HBV management to improve patient outcomes. Most studies strongly recommend complete alcohol abstinence. However, the question of how much alcohol is truly safe for HBV patients remains unresolved, warranting further research to define clear consumption limits.

## 3 Alcohol-induced hepatocyte injury and its role in HBV pathogenesis and HCC risk

### 3.1 Alcohol metabolism and its shift during chronic alcohol use

About 90-98% of ingested alcohol is metabolized in the liver, making it the primary organ responsible for alcohol breakdown and elimination. The remaining 2-10% is excreted unchanged through urine, sweat, and breath (14). Cells outside the liver, such as neurons, astrocytes, gastrointestinal epithelial cells, lung cells, and immune cells, including monocytes and lymphocytes, can metabolize small amounts of alcohol using enzymes like cytochrome P450 (CYP2E1) and catalase. These extrahepatic pathways contribute to less than 10% of overall alcohol metabolism.

Alcohol metabolism primarily involves a two-step enzymatic process catalyzed by alcohol dehydrogenase (ADH) and aldehyde dehydrogenase (ALDH), converting ethanol first into acetaldehyde and then into acetate. Later is further broken down into carbon dioxide and water. The liver plays a central role in clearing acetaldehyde, a highly reactive and carcinogenic intermediate (15). In chronic alcohol use, sustained acetaldehyde accumulation exposes hepatocytes to toxic concentrations. Blood acetaldehyde can transiently reach 10-20 µM in the portal circulation immediately after alcohol intake, while hepatic acetaldehyde concentrations may be 5–10 times higher (16). Exposing hepatocytes to such high levels is sufficient to induce protein adduct formation, oxidative stress, and DNA damage (17, 18).

In addition, chronic alcohol intake shifts metabolism toward CYP2E1 because ADH becomes saturated at higher alcohol levels. This leads to strong induction and stabilization of CYP2E1 in the liver and other tissues. The shift increases alcohol breakdown but also generates large amounts of reactive oxygen species (ROS), contributing to tissue damage during chronic drinking. The process is dynamic and reversible upon alcohol withdrawal. Factors such as alcohol-mediated induction of CYP2E1, ALDH2 deficiency, and impaired liver function can further elevate acetaldehyde levels and reduce its clearance.

Alcohol metabolism disrupts cellular homeostasis through interconnected mechanisms that impact multiple organelles and signaling pathways. Its toxic byproducts, acetaldehyde and malondialdehyde, react with proteins to form malondialdehyde-acetaldehyde (MAA) adducts. This distorts protein structure and impairs endoplasmic reticulum (ER) function. Acetaldehyde further compromises the ER by modifying chaperones and enzymes, interfering with disulfide bond formation and glycosylation, depleting glutathione, disturbing calcium signaling, and causing homocysteine accumulation (19–21). These combined disruptions overwhelm the ER protein-folding capacity, triggering the unfolded protein response (UPR) and promoting inflammation and apoptosis.

Alcohol and endogenous aldehydes also cause DNA double-strand breaks and chromosomal rearrangements in hematopoietic stem cells. These genetic damages compromise stem cell function, promote abnormal mutations, and increase the risk for cancer and blood disorders (18). Mitochondrial metabolism is similarly affected. Alcohol increases the NADH/NAD^+^ ratio, reduces ATP production, and disrupts fatty acid oxidation, leading to steatosis and mitochondrial injury (22, 23). The resulting redox imbalance and loss of antioxidant defenses heighten susceptibility to oxidative stress. Alcohol additionally activates stress pathways such as MAPK, NF-κB, and JNK, further driving inflammation and cell death (24, 25).

By weakening the intestinal barrier, alcohol allows bacterial lipopolysaccharide (LPS) to reach the liver and activate TLR4 signaling in Kupffer cells, amplifying hepatic inflammation (26, 27). In addition, alcohol directly activates Kupffer cells, monocytes, macrophages, and antigen-presenting cells, altering cytokine production and modulating T-cell responses (28–30). These collectively promote the immune dysregulation, persistent inflammation, and tissue injury characteristic of ALD.

### 3.2 Alcohol’s role in HBV pathogenesis

In HBV-infected individuals, alcohol further enhances liver damage by promoting viral replication and impairing immune responses, which contributes to increased hepatitis activity. For example, studies from our lab and others show that alcohol increases HBV DNA and viral protein levels, correlating with higher ALT/AST and liver injury (31–33). This has been seen in both immunocompetent and immunocompromised HBV transgenic mouse models. In these models, alcohol-fed mice exhibit multiple-fold increases in HBV surface antigen (HBsAg), serum viral DNA, HBV RNA, and liver viral protein expression. This results in severe hepatic steatosis, liver injury, and eventually progresses to end-stage liver disease (31, 33, 34).

A key mechanism underlying this effect is that alcohol enhances HBV replication by stimulating the activity of the viral core and surface promoters. This effect is strongly amplified by CYP2E1-mediated oxidative stress, which increases transcription factor activity on these promoters. Importantly, this oxidative effect can be reversed by antioxidants such as glutathione (35). This underscores oxidative stress as a central driver in alcohol-induced augmentation of HBV replication, alongside alcohol metabolism impacts on immune impairment. In addition, alcohol can alter the lipid composition and function of lipid rafts. These rafts are specialized domains within hepatocyte membranes that are required for HBV entry, assembly, and release. Changes in lipid rafts enhance HBV entry into liver cells and may facilitate virus assembly and release, thereby indirectly increasing viral replication (36).

### 3.3 Alcohol as a driver of HBV progression to HCC

HBV promotes HCC through multiple mechanisms, including persistent inflammation, viral DNA integration, and epigenetic dysregulation (37). Viral proteins such as HBx and preS/S contribute to oxidative stress and activate oncogenic pathways. Alcohol exacerbates these effects by enhancing viral replication, liver damage, and fibrosis, collectively accelerating hepatocarcinogenesis. A key mechanism underlying this synergy involves alcohol-induced activation of LPLA2 via ATF4-dependent transcription, thereby elevating bis(monoacylglycero)phosphate (BMP) levels in tumors. LPLA2 is a lysosome-associated enzyme central to lipid metabolism. LPLA2 drives HCC cell proliferation by activating the MAPK/ERK signaling cascade, which is further strengthened by BMP synthase CLN5 and BMP supplementation.

Knockdown of LPLA2 or CLN5 markedly reduces alcohol-enhanced tumor growth. Clinically, high LPLA2 expression correlates with poor patient outcomes, underscoring the ATF4/LPLA2/BMP axis as a pivotal driver of alcohol-accelerated HBV-associated HCC (38). HBV proteins, including HBx and HBc, are most affected by alcohol metabolism, mediating many downstream effects linking alcohol use with worsened HBV-related liver disease. HBx aggravates alcohol-induced liver injury by increasing oxidative stress, lipid peroxidation, and harmful lipid changes (39). In HBx-transgenic mice, alcohol causes higher acetaldehyde levels and more severe steatohepatitis by allowing HBx to bind and degrade mitochondrial ALDH2, reducing acetaldehyde clearance. The resulting acetaldehyde buildup drives oxidative stress, toxic lipid production, and cancer risk (40).

Studies in both humans and mice indicate that ALDH2 deficiency leads to greater susceptibility to carcinogen-induced liver tumors. ALDH2 deficiency did not affect liver disease progression but increased HCC risk in cirrhotic HBV patients with AUD (41). Mechanistically, ALDH2-deficient hepatocytes produce large quantities of oxidized mitochondrial DNA, which are packaged into extracellular vesicles and transferred to neighboring cancer cells. Together with acetaldehyde, this activates several oncogenic pathways, including JNK, STAT3, BCL-2, and TAZ. These combined signals promote HCC development and progression (41).

Additionally, reduced ALDH2 expression in liver cancer tissues correlates with increased tumor growth, migration, and poor prognosis, and this deficiency disrupts cellular redox balance and metastatic potential (42–44). HBx also modulates microRNAs (including miR-187-5p and miR-3188), interferes with tumor suppressors, and stimulates stem-like properties via pathways such as Wnt/β-catenin and CXCR4. It enhances hepatocyte proliferation, migration, invasion, cytoskeletal remodeling, and lipid dysregulation, further promoting liver injury and carcinogenesis (45–48). By disrupting cell signaling, inhibiting DNA repair, altering gene expression, and reprogramming metabolism, HBx induces genomic instability, chronic inflammation, stemness, and abnormal lipid metabolism. This accelerates progression to cancer and can be particularly pronounced in CHB infection, where persistent viral protein expression and ongoing liver injury create a pro-oncogenic environment.

Accumulation of high levels of HBsAg adds another layer of oncogenic stress by inducing ER stress. This initially activates autophagy but eventually impairs its degradation phase. ER stress-related transcription factors ATF4 and ATF6 suppress LAMP2, essential for autophagosome-lysosome fusion, leading to impaired autophagic flux and cellular waste buildup. This sustains hepatocyte proliferation and advances HCC development (49). Furthermore, the HBV PreS1 protein promotes HCC by stimulating the emergence and self-renewal of liver cancer stem cells. It enhances cancer stem cell marker expression, promoting tumor growth and supporting a highly proliferative, drug-resistant cell population that drives cancer progression and recurrence (50).

Collectively, these findings highlight the synergistic harmful impact of alcohol and HBV on liver disease progression, consistent with epidemiological data showing higher HBV marker levels and worse liver outcomes, including cirrhosis and HCC, in alcoholic individuals with HBV infection.

## 4 Immune dysregulation at the intersection of alcohol use, HBV, and HCC

HBV is largely noncytopathic, meaning it does not directly kill hepatocytes. Instead, liver injury arises primarily from the host immune response against HBV-infected cells. Cytotoxic CD8+ T lymphocytes (CTL), together with other immune cells and mechanisms, target infected hepatocytes, leading to apoptosis and necrosis, which drive liver inflammation and damage. Over time, this immune-mediated injury can progress to fibrosis, cirrhosis, and HCC. Alcohol consumption exacerbates this process by synergistically impairing both innate and adaptive immune responses critical for controlling HBV. Excessive alcohol intake suppresses natural killer (NK) cell activity, reduces T and B cell populations, disrupts antigen presentation via the major histocompatibility complex class I (MHC-I) pathway, and diminishes production of antiviral cytokines, including interferons (IFN-α/β and IFN-γ). Collectively, these effects facilitate persistent HBV replication, elevate viral load, and impair viral clearance. This leads to sustained liver inflammation and accelerates progression toward HCC.

### 4.1 Alcohol interference with HBV MHC-I antigen display

Alcohol and acetaldehyde impair HBV antigen presentation by disrupting multiple steps of the MHC-I pathway in hepatocytes. Acetaldehyde reduces the surface display of HBV peptide-MHC-I complexes by suppressing proteasome activity, downregulating immunoproteasome subunits (LMP2, LMP7), and impairing peptide-loading components such as TAP1 and tapasin, while also inhibiting IFN-γ signaling (51–53). In parallel, chronic ethanol exposure in mice impairs dendritic cell presentation of exogenous antigens through MHC II and alters costimulatory profiles, leading to diminished antigen−specific T−cell activation despite largely preserved MHC I cross−presentation (54). Additionally, acetaldehyde-induced ER stress and Golgi fragmentation interfere with the trafficking of these complexes to the cell surface, further limiting recognition by CTLs (55, 56).

Efficient MHC I antigen display depends on intact anterograde ER-to-Golgi-to-plasma membrane transport of peptide-loaded complexes (57). In alcohol-exposed hepatocytes, acetaldehyde-driven ER stress and Golgi fragmentation impair this secretory pathway, leading to intracellular retention of MHC I complexes within the ER, ER-Golgi intermediate compartment (ERGIC), or fragmented Golgi (55, 58–60). This trafficking blockade directly reduces surface presentation of HBV peptide-MHC I complexes, as demonstrated in HBV models in which acetaldehyde selectively suppresses MHC I surface expression through organelle dysfunction. Alcohol metabolism also diminishes proteasomal and aminopeptidase activity, reducing peptide generation required for MHC-I loading (61). Collectively, these mechanisms compromise antigen processing, presentation, and immune recognition of HBV-infected hepatocytes, promoting viral persistence and immune evasion.

### 4.2 T cells

Alcohol impairs immune recognition of HBV-infected hepatocytes by reducing their ability to present CTL epitopes. Clinical studies in HBV-positive patients with alcohol use show distinct immune alterations depending on disease stage. In compensated cirrhosis, T cells display both activation and exhaustion markers, accompanied by reduced cytokine production. In decompensated cirrhosis, CD8^+^ T cell numbers are decreased, CD4/CD8 ratios are altered, and T cell proliferation increases, but activation and exhaustion markers are lost. These changes occur within a highly inflammatory environment. Similar immune defects are observed in alcohol-related cirrhosis, including depletion and functional impairment of MAIT (mucosal-associated invariant T) cells and elevated soluble immune checkpoint molecules, such as sTIM-3 (62). Together, these alterations contribute to weakened antiviral immunity and create a permissive environment for viral persistence.

In our study, we utilized fumarylacetoacetate hydrolase (Fah)-/-, Rag2-/-, common cytokine receptor gamma chain knock-out (FRG-KO) humanized mice engrafted with HLA-A2-positive hepatocytes. Alcohol feeding resulted in decreased presentation of the HBV core 18–27 epitope bound to HLA-A2 on infected hepatocytes. This reduction stemmed from alcohol-induced suppression of proteasome activity and ER stress, which collectively impaired HBV peptide processing and inhibited trafficking of HBV-MHC-I complexes to the cell surface. The resulting attenuation of MHC class I-restricted antigen presentation weakened CTL-mediated cytotoxicity against infected hepatocytes, facilitating viral persistence and progression to end-stage liver disease (63).

Chronic alcohol exposure also disrupts hepatic lipid metabolism during HBV infection. This occurs through activation of the HBx/SWELL1/arachidonic acid signaling axis, with HBx and SWELL1 upstream and arachidonic acid as a downstream effector. Activation of this pathway disturbs lipid synthesis and breakdown, causing abnormal lipid accumulation and metabolic dysfunction in hepatocytes (64). HBx further contributes to hepatocarcinogenesis by modulating genes involved in arachidonic acid metabolism (65). Additionally, alcohol and HBV co-exposure enhances regulatory T-cell (Treg) activation in HBV-transgenic mice, leading to immunosuppression that further limits the clearance of infected hepatocytes (64).

CHB is also characterized by T-cell exhaustion, which is worsened by alcohol and includes up-regulation of PD-1, CTLA-4, and TIM-3 (66), impaired mitochondrial function in T cells (23, 67), and impaired antigen-presenting function of dendritic and Kupffer cells (68–70). This promotes the persistence of HBV-infection, development of end-stage liver diseases, including HCC. Even moderate drinking increases the risk of HCC progression.

Paradoxically, controlled low-dose alcohol exposure enhances dendritic cell (DC) maturation and exosome-driven T-cell activation in CHB, countering typical immunosuppression. *In vitro* alcohol treatment of DCs from HBV patients and transgenic mice upregulates costimulatory molecules (CD80/CD86), MHC, and T-cell priming cytokines, restoring functional DC responses. Moreover, exosomes from these alcohol-conditioned DCs boost HBV-specific CD8^+^ and CD4^+^ T-cell proliferation and effector function *in vivo*, challenging the notion of alcohol as solely detrimental by revealing context- and dose-dependent immunostimulatory effects (71).

Collectively, these findings indicate that chronic or high-dose alcohol consumption profoundly impairs antigen presentation, CTL function, and immune-metabolic homeostasis in the liver, thereby promoting viral persistence, immune evasion, and metabolic stress. These combined effects create a permissive environment for progressive liver disease and increase the risk of HBV-associated HCC.

### 4.3 B cells

Alcohol reduces both the number and function of intrahepatic B cells, thereby decreasing antibody production against HBV antigens. This impairment of B-cell-mediated immunity delays viral clearance and promotes CHB infection. Mechanistically, alcohol increases Fas expression on intrahepatic B220^+^ and CD138^+^ B cells and stimulates production of immunosuppressive cytokines, including TGF-β and IL-10, by CD19^+^ B cells. Alcohol also decreases intrahepatic T peripheral helper (Tph) cells and T follicular helper (Tfh) cells, lowers IL-21 production by Tfh-like and CD8^+^PD-1^+^ cells, and reduces splenic germinal center B cell areas. Since Tph and Tfh cells support B cell differentiation and antibody production, their reduction impairs humoral immune responses, further hindering HBV clearance (72).

### 4.4 NK cells

NK cells are key innate immune effectors in HBV infection, where they control viral replication through cytolysis, degranulation, and secretion of antiviral cytokines such as IFN-γ and TNF-α (73, 74). These early NK-driven responses help limit HBV spread but can also cause liver injury by killing infected hepatocytes through Fas/FasL and TRAIL pathways (74). In CHB infection, NK cells exhibit a dual role: they continue to suppress viral replication through non-cytolytic mechanisms, yet their heightened cytotoxicity contributes to inflammation, fibrosis, and progressive liver damage (75, 76).

Alcohol exposure further disrupts NK cell function in HBV-infected livers. Alcohol reduces NK antiviral activity, alters activating receptor expression, and creates an immunosuppressive cytokine environment that weakens early HBV clearance (77, 78). At the same time, alcohol can drive inappropriate NK activation that worsens liver injury. In HBV-carrier mouse models, alcohol-exposed NK cells suppress CD8^+^ T-cell antiviral responses, promoting T-cell dysfunction and delayed viral control. NK-cell depletion restores this CD8^+^ T-cell activity (79). Alcohol amplifies NK-cell immune dysregulation by weakening their antiviral activity while enhancing their tissue-damaging responses. This dual effect accelerates HBV persistence and worsens liver disease progression.

### 4.5 Macrophages

Macrophages sense HBV infection mainly through pattern recognition receptors (PRRs) such as TLR4, TLR2, and other DNA sensors like cGAS-STING that detect viral components, HBV DNA, HBsAg, and core proteins (80). The recognition of HBV by these receptors activates downstream signaling pathways, including the NF-κB and JAK-STAT pathways. NF-κB promotes the transcription of pro-inflammatory cytokines (IL-1β, TNF-α). At the same time, the JAK-STAT pathway, triggered by interferons, induces interferon-stimulated genes (ISGs) with antiviral effects. These processes allow macrophages to initiate innate immune responses against HBV (80, 81). In our study, IFN-α-activated macrophages suppressed HBV replication in hepatocytes by decreasing HBV RNA, DNA, and cccDNA levels via induction of ISGs such as APOBEC3G, ISG15, and OAS1. Alcohol disrupts this protective communication between macrophages and hepatocytes, inhibiting ISG induction and increasing HBV replication, which weakens innate antiviral immunity (33).

Recent single-cell RNA sequencing (scRNA-seq) studies have further clarified macrophage heterogeneity and functional states in the context of HBV infection and alcohol exposure. Comparative single-cell analyses of liver immune cells from patients with alcoholic liver cirrhosis (ALC) and HBV-related cirrhosis (HBV-LC) reveal etiology-specific macrophage and broader immune programs. In ALC, intrahepatic monocytes/macrophages are markedly expanded while adaptive immune cells, particularly B and plasma cells, are reduced and metabolically altered, consistent with alcohol-driven myeloid dominance and impaired humoral immunity. In HBV-LC, macrophages are relatively less expanded and appear functionally distinct, with immune profiles more compatible with chronic virus-driven activation rather than the pronounced innate skewing observed in ALC (82). Despite their central role in immune sensing, cytokine production, and hepatocyte communication, few studies have directly examined the combined effects of alcohol, hepatic macrophages, and HBV, limiting mechanistic understanding of how alcohol accelerates viral persistence and disease progression.

More broadly, chronic alcohol exposure reshapes hepatic macrophage populations by promoting the recruitment of bone marrow-derived monocytes, resulting in a mix of resident Kupffer cells and infiltrating macrophages. These macrophages can adopt pro-inflammatory M1-like states that worsen liver injury or shift toward M2-like repair phenotypes (83–85). Alcohol-induced gut permeability further raises LPS levels and activates macrophages through TLR4. This activation leads to the production of inflammatory cytokines such as TNF-α and IL-1β, which contribute to liver inflammation and fibrosis (86). Hepatocyte-macrophage crosstalk also amplifies alcohol-related inflammation. Alcohol stimulates macrophage activation via caspase-dependent release of CD40L-containing extracellular vesicles (EVs) from hepatocytes, which promotes inflammation and contributes to ALD (87).

Additionally, macrophage-derived MLKL supports phagocytosis, aiding bacterial clearance and controlling liver inflammation. In ALD, loss or dysfunction of MLKL in macrophages impairs this protective function, leading to heightened inflammation, tissue damage, and worsened outcomes (88). Chemical models of HCC have demonstrated a direct link between alcohol consumption, macrophage activation, and increased HCC risk. For example, chronic alcohol feeding in diethylnitrosamine (DEN)-induced HCC mice leads to reduced antitumor CD8+ T cells and an increase in tumor-associated macrophages (TAMs), which tend to polarize toward the M2 phenotype after alcohol exposure (89).

This M2 polarization is associated with increased fibrosis and epithelial-mesenchymal transition (EMT), creating a pro-tumor microenvironment that promotes tumor growth. Moreover, alcohol consumption induces macrophages to produce IL-6 via a PRMT1-dependent mechanism, which activates STAT3 signaling in hepatocytes and drives tumor progression. PRMT1, a key enzyme mediating arginine methylation, influences macrophage polarization toward the M2 anti-inflammatory phenotype that supports liver tumor promotion (90). Together, these findings highlight how alcohol modulates macrophage functions at multiple levels to exacerbate liver disease and HCC development.

**Figure 1:**
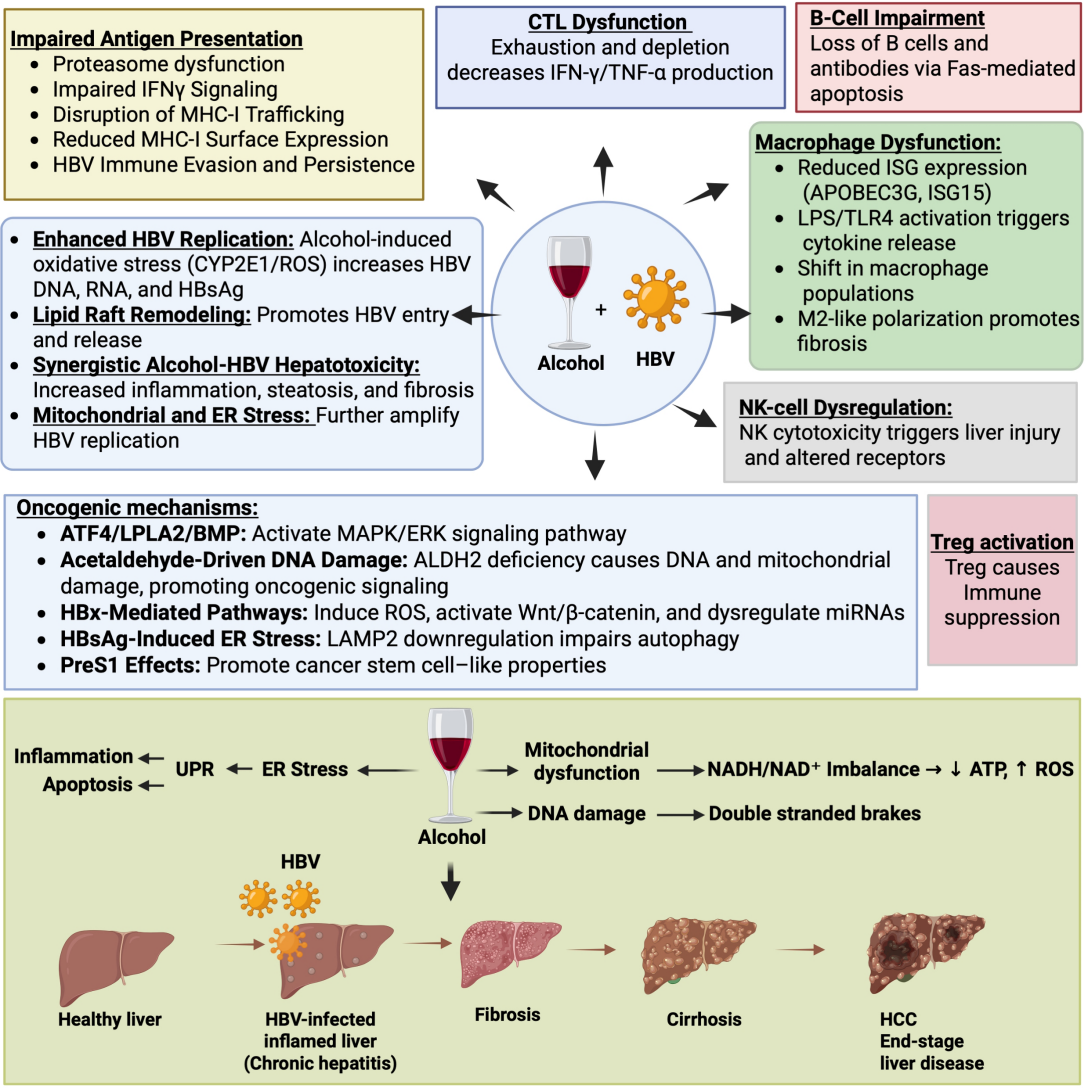
Intersecting mechanisms by which alcohol exacerbates CHB infection and accelerates liver disease progression. This figure illustrates the multifaceted ways by which alcohol interacts with HBV infection to impair antiviral immunity, promote viral persistence, and worsen liver injury. Impaired antigen presentation (top left): Alcohol disrupts proteasome activity, TAP1/tapasin function, IFN-γ signaling, and induces ER/Golgi fragmentation, collectively reducing MHC-I restricted antigen display on hepatocytes. This impairs CTL recognition of HBV-infected cells. T-cell dysfunction (top center): Chronic alcohol exposure aggravates HBV-driven T-cell exhaustion, characterized by reduced numbers and impaired function of antiviral CD8^+^ T cells (CTLs), decreased IFN-γ and TNF-α production, and diminished antiviral clearance. B-cell impairment (top right): Alcohol reduces B-cell abundance and antibody production and enhances Fas-mediated apoptosis, weakening humoral immunity against HBV. Macrophage dysfunction (right): Alcohol downregulates ISGs (e.g., APOBEC3G, ISG15), activates LPS/TLR4 signaling, and alters liver macrophage populations, promoting Kupffer cell dysfunction, infiltration of pro-inflammatory macrophages, and M2-like tumor-associated macrophage polarization. Hepatocyte-derived extracellular vesicles further activate macrophages, amplifying cytokine release and inflammation. NK-cell dysregulation (right): Alcohol alters NK-cell receptor expression and cytotoxicity, contributing to hepatic injury and impaired antiviral defense. Viral and metabolic interactions (left center): Alcohol enhances HBV replication via CYP2E1-derived ROS, increased HBV DNA/RNA/HBsAg production, elevated core/surface promoter activity, and alcohol-induced lipid changes that facilitate HBV entry and virion release. This triggers systemic inflammation, steatosis, mitochondrial dysfunction, and ER stress that accelerate hepatocyte injury during co-exposure. Oncogenic mechanisms (lower left): Alcohol and HBV cooperatively drive carcinogenesis through multiple pathways, including ATF4-dependent LPLA2/BMP/MAPK/ERK activation; acetaldehyde-mediated oxidative DNA damage via ALDH2 deficiency; HBx-induced ROS, Wnt/β-catenin signaling, and miRNA dysregulation; HBsAg-induced ER stress with LAMP2-mediated impairment of autophagy; and PreS1-associated oncogenic stress. Treg activation (lower right). Activation of regulatory Tregs suppresses antiviral and antitumor immune responses, facilitating immune evasion and persistent inflammation. Bottom panel: Diagram summarizing stepwise disease progression from healthy liver to CHB infection, fibrosis, cirrhosis, and HCC. Alcohol amplifies mitochondrial dysfunction, ER stress, DNA damage, ROS accumulation, NADH/NAD^+^ imbalance, ATP depletion, and genomic instability (e.g., double-stranded DNA breaks), collectively accelerating progression toward end-stage liver disease.

While direct evidence linking alcohol, macrophage dysfunction, and HBV-driven HCC remains limited, alcohol-induced macrophage activation and altered cytokine production likely exacerbate HBV persistence and liver injury. Evidence from alcohol-related and chemical HCC models suggests that alcohol may polarize macrophages toward a pro-tumorigenic M2-like phenotype. This suppresses antiviral immunity and promotes a liver microenvironment that enhances HBV persistence and hepatocarcinogenesis, accelerating both liver disease and tumor progression. **Figure 1** summarizes the effects of alcohol on HBV-mediated liver damage and HCC.

## 5 Non-alcoholic cofactors contributing to HBV-associated HCC

Alcohol can be viewed as one of several key cofactors that modify the natural history of CHB infection, alongside metabolic syndrome, obesity, and viral coinfections. Importantly, these factors can promote liver disease progression both independently of alcohol and in synergy with it, converging to accelerate fibrosis and hepatocarcinogenesis. Metabolic syndrome drives insulin resistance, chronic low-grade inflammation, and lipotoxicity, thereby enhancing hepatocyte proliferation and genomic instability on top of HBV-driven oncogenic pathways. In a large Chinese cohort of patients with CHB infection, metabolic syndrome was independently associated with an approximately two-fold increased risk of HCC after adjustment for age, sex, smoking, alcohol use, cirrhosis, and AST levels (91). Consistently, another retrospective cohort study from China demonstrated that the presence of metabolic dysfunction-associated steatotic liver disease (MASLD) is associated with a higher risk of HCC in patients with CHB (92). Both studies adjusted for alcohol consumption as a standard covariate in multivariable models alongside age, sex, smoking, cirrhosis, and AST to isolate metabolic effects. This approach reflects general CHB populations with heterogeneous drinking levels rather than alcohol-selected subgroups.

Beyond Asian cohorts, European data further support the independent contribution of metabolic and cardiovascular comorbidities to adverse HBV outcomes. CHB patients with obesity, diabetes, metabolic syndrome, or cardiovascular disease exhibit markedly higher risks of cirrhosis and HCC, reflecting accelerated inflammation and fibrosis even in the setting of antiviral HBV control (93). A European cohort from Erasmus MC (Netherlands) showed that overweight, diabetes, hypertension, and dyslipidemia independently increased liver-related events, including HCC, transplantation, and mortality, with multiple comorbidities amplifying risk several-fold across subgroups, including virally suppressed and non-cirrhotic patients (94). Data from the Danish DANHEP registry similarly demonstrated that CHB patients with cardiometabolic disorders experience higher rates of early liver complications compared with those without such conditions, underscoring the substantial burden of metabolic comorbidity in European HBV populations (95).

In the United States (US), studies highlight the contribution of demographic and clinical factors to HCC risk. A US-based CHB cohort identified increasing age and male sex as strong predictors of HCC. Patients with HCC more frequently exhibited comorbidities and risk factors such as tobacco use, recreational drug use, anemia, ascites, portal hypertension, chronic kidney disease, and co-infection with HCV (96). North American studies, including the North American AIDS Cohort Collaboration on Research and Design (NA−ACCORD), demonstrate that HBV-HIV coinfection markedly elevates HCC risk compared with either infection alone, primarily through faster progression of liver fibrosis and cirrhosis and uncontrolled HBV replication (97). Data from the HIV/AIDS Cancer Match (HACM) study (2001-2019) show that HIV patients (including HBV-coinfected subsets) faced 2.8-fold higher HCC rates than the general population, though declining over time due to better viral control (98).

Overall, these findings underscore that metabolic, cardiovascular, and other comorbid conditions significantly amplify HCC risk in CHB infection, both independently of alcohol and in combination with it. This highlights the need for vigilant monitoring and risk stratification across all patient subgroups.

## 6 Perspective: The intersecting burdens of HBV infection, alcohol use, and liver cancer

While the oncogenic mechanisms of HBV, including viral DNA integration, chronic liver inflammation, and HBV protein-driven signaling, are well characterized, growing evidence highlights alcohol consumption as a potent and often underrecognized cofactor linked to disease progression. Numerous studies demonstrate that the combination of HBV infection and chronic alcohol use markedly increases the risk of cirrhosis, liver failure, and HCC. Despite these advances, notable gaps and unresolved questions persist.

For instance, research on individuals with ALD with or without HBV faces multiple methodological challenges. Alcohol intake is commonly self-reported, resulting in underestimation. Studies vary in their definitions of “moderate” versus “heavy” drinking as well as diagnostic criteria for ALD, cirrhosis, and HCC (e.g., imaging, biopsy, or registry data). Observational cohorts are further confounded by smoking, obesity, socioeconomic status, healthcare access, HBV viral load, antiviral treatment, and other hepatotoxic exposures. Moreover, because liver disease and cancer typically develop over decades, many studies lack the long-term follow-up needed to establish clear temporal links between alcohol use, HBV, and HCC. This makes it challenging to quantify the synergistic effects of these risk factors fully.

Another limitation is that many studies examining alcohol use, HBV infection, and HCC have been conducted primarily in East Asian populations (Taiwan, Korea, China, and Japan). These regions have high HBV endemicity and distinct drinking patterns that differ from those in other parts of the world. This concentration may limit the generalizability of findings, as epidemiology, risk factors, and healthcare access vary geographically. Genetic variations in alcohol metabolizing ADH and ALDH enzymes also differ globally. For example, ADH1B and ALDH2 gene variants are common in 30-50% of East Asians, influencing faster or slower alcohol metabolism and acetaldehyde clearance, but are rare in Europeans and Africans (99).

In East Asia, the predominance of HBV genotype C (high oncogenic potential) and the ALDH2*2 polymorphism (associated with alcohol flush and altered drinking behavior) further amplifies the interaction between alcohol and HBV (100–102). By contrast, HBV genotypes A and D dominate in Western populations and genotype E in Africa (103, 104). Additional regional factors such as high baseline HBV endemicity (5-18%), dietary aflatoxin exposure, and lower NAFLD prevalence further limit direct extrapolation (105). In Western populations, alcohol appears less synergistic with HBV, while HCV infection and alcohol are more prominent drivers of HCC, whereas synergy with HBV has been observed in African contexts. This highlights the need for region-specific risk models.

​Furthermore, Indian populations exhibit different variant profiles, such as the ADH3*1/*1 genotype in North Indians, associated with increased risk of alcohol-related pancreatitis (106). Variants in genes related to oxidative stress response (GSTM1, GSTT1, MnSOD, CYP2E1) show diverse associations with alcoholic liver disease across populations, making it difficult to generalize findings. Moreover, the ability of the immune system (mainly CTLs) to recognize HBV-infected hepatocytes is substantially limited by alcohol exposure at the level of MHC-I restricted antigen presentation on target hepatocytes (63). The prevalence of HBV genotypes for MHC-class I restricted HBV presentation differs across geographical regions (104).

Mechanistically, while alcohol-induced oxidative stress, immune suppression, and activation of HBV transcription have been demonstrated, the complete molecular pathways remain incompletely understood. Standard experimental animal models, such as mice, are naturally resistant to HBV infection and fail to fully replicate the complex interactions between alcohol and HBV observed in humans. Although humanized mouse models, which incorporate human hepatocytes and sometimes immune cells, offer a valuable platform to study human-specific viral-host interactions and immune responses, they also have significant limitations. These include high costs, variability in the quality and engraftment efficiency of human liver tissue, the limited lifespan of human hepatocytes in the mouse liver, and incomplete reconstitution of the human immune system.

Additionally, low levels of viral replication and insufficient immune-mediated liver pathology often limit their ability to fully mimic CHB infection and alcohol-driven liver disease progression seen in humans. These limitations make it challenging to develop a comprehensive mechanistic map of how alcohol affects HBV persistence, immune evasion, viral replication, and oncogenic transformation. As a result, progress in designing targeted therapies and effective management strategies is hindered.

In conclusion, the combination of HBV infection and alcohol use significantly worsens liver disease and increases cancer risk. However, research is limited by population differences, methodological challenges, and incomplete mechanistic understanding. Future studies should employ standardized definitions, extended follow-up, diverse populations, and enhanced experimental models. Such approaches will improve our understanding of, and ability to mitigate, the combined impact of alcohol and HBV on liver disease and cancer risk.
